# Factors associated to the duration of COVID-19 lockdowns in Chile

**DOI:** 10.1038/s41598-022-13743-8

**Published:** 2022-06-09

**Authors:** Jessica Pavani, Jaime Cerda, Luis Gutiérrez, Inés Varas, Iván Gutiérrez, Leonardo Jofré, Oscar Ortiz, Gabriel Arriagada

**Affiliations:** 1 Faculty of Mathematics, Pontificia Universidad Católica de Chile, Av. Vicuña Mackenna 4860, 7810000 Macul, Chile; 2 Faculty of Medicine, Pontificia Universidad Católica de Chile, Santiago, Chile; 3 ANID-Millennium Science Initiative Program-Millennium Nucleus Center for the Discovery of Structures in Complex Data, Santiago, Chile; 4 School of Engineering, Pontificia Universidad Católica de Chile, Santiago, Chile; 5 Unit of Epidemiology, Institute of Agri-Food, Animal and Environmental Sciences, Universidad de O’Higgins, Ruta 90 km3, San Fernando, Chile

**Keywords:** Infectious diseases, Epidemiology

## Abstract

During the first year of the COVID-19 pandemic, several countries have implemented non-pharmacologic measures, mainly lockdowns and social distancing, to reduce the spread of the SARS-CoV-2 virus. These strategies varied widely across nations, and their efficacy is currently being studied. This study explores demographic, socioeconomic, and epidemiological factors associated with the duration of lockdowns applied in Chile between March 25th and December 25th, 2020. Joint models for longitudinal and time-to-event data were used. In this case, the number of days under lockdown for each Chilean commune and longitudinal information were modeled jointly. Our results indicate that overcrowding, number of active cases, and positivity index are significantly associated with the duration of lockdowns, being identified as risk factors for longer lockdown duration. In short, joint models for longitudinal and time-to-event data permit the identification of factors associated with the duration of lockdowns in Chile. Indeed, our findings suggest that demographic, socioeconomic, and epidemiological factors should be used to define both entering and exiting lockdown.

## Introduction

At the beginning of 2020, when the World Health Organization (WHO) declared COVID-19 a pandemic, countries started to implement containment measures to reduce viral transmission, mainly lockdowns and social distancing. Rapidly, more than half of the global population was under strict forms of movement restrictions and social distancing. The strategies adopted by national governments varied widely, and their efficacy has been investigated. Examples of these are the studies conducted by Alfano and Ercolano^1^, who studied the efficacy of lockdown measures in 202 countries, proving that, on average, lockdown reduces COVID-19 contagiousness, and its efficiency starts after approximately 3 weeks. On the other hand, a study conducted by Di Domenico et al.^2^ focused on lockdown exit strategies. The authors pointed out that lifting lockdowns without an exit strategy would lead to large rebound effects. In addition, Onyeaka et al.^3^ highlighted some of the more significant impacts of the lockdown over the world.

In Chile, the first epidemic control measures were kindergarten and school closing, followed by country borders closure. A few days later, on March 25th, the government implemented localized lockdowns at the commune level, the smallest administrative division in Chile. Lockdowns were established by the government based on several criteria defined by the Chilean Ministry of Health. Four indicators were considered: the total number of active cases, the increment in the incidence of active cases, the total number of active cases per km^2^, and the availability of beds in intensive care units (ICU), all measured per commune (see the follow section for more details about Chilean administrative division).

The Ministry of Health also established criteria for ending lockdowns. At a regional level, the occupancy of the ICU system should be less than or equal to 85%, and the percentage of positivity of the polymerase chain reaction (PCR) exams less than 10% in the previous seven days. At the commune level, a steady decline in new cases during the previous 21 days was required, which means the effective reproductive number was less than or equal to one. In addition, there should be a capacity to track and isolate 90% of new confirmed cases in less than 48 h, together with the capacity of identifying and tracking 75% of contacts of those cases for 14 days.

Although the Chilean government established the criteria to start and end a lockdown, there is still no strong scientific evidence to support them; thus, they could be arguable. Motivated by this concern, the main objective of this study was to explore demographic, socioeconomic, and epidemiological factors associated with the duration of localized lockdown in Chile. To pursue our objectives, we used joint models for longitudinal and time-to-event data, which considered the number of days of lockdown for each Chilean commune and the longitudinal information jointly.

## Material and methods

### Study location

Localized in South America, Chile occupies a long and narrow coastal strip between the Andes Mountains range and the Pacific Ocean. It borders Peru to the north, Bolivia to the northeast, and Argentina to the east. The country is divided into 16 regions, which are the first-level administrative division of the country. Each region is divided into provinces, which are the second-level administrative division, resulting in 56 provinces. The third level of the administrative division is the communes (or municipalities), totaling 346 communes, of which 147 were under lockdown during the study period; thus, they were considered in this study.

According to the Chilean National Institute of Statistics, the estimated country population was 19,458,173 inhabitants in 2020, much of it concentrated in the Metropolitan Region, whose capital city is Santiago. Regarding the communes, only 55 of 346 have more than 100,000 inhabitants, with the median population being 18,546 inhabitants (minimum 138, maximum 646,000). Chile is among the largest economy in Latin America; however, despite its economic progress and poverty reduction over the last few decades, the country has a Gini coefficient of 0.44, which represents a high social inequality. As elsewhere, the COVID-19 pandemic has severely impacted the Chilean economy. According to the World Bank^4^, the Chilean per capita gross domestic product decreased 5.8% between 2019 and 2020.

### Study design

Factors associated with the duration of localized lockdowns during the COVID-19 pandemic in Chile were assessed using a retrospective cohort study design^5^. In this design, researchers select a group of study units (i.e., commune under lockdown) in such a way that they have been exposed to different levels of certain predictors, and then follow them retrospectively over time to record the occurrence or not of a predefined event. In this particular investigation, the main outcome corresponds to the time elapsed until the end of the lockdown (i.e., the lockdown duration). No formal sampling of communes under lockdown was performed since all lockdowns implemented between March 25th and December 25th, 2020 were included in this study. All methods were performed in accordance with relevant guidelines and regulations.

### Outcomes

In follow-up studies, two types of data can be observed: information collected over time (longitudinal data) and time until an event of interest occurs (survival data). In this study, both were included as outcomes. For the time-to-event analysis, we considered the number of days a commune was under lockdown; to do so, we recorded the number and duration of lockdowns implemented in Chile between March 25th and December 25th, 2020. If a commune was still under lockdown on December 25th, it was considered a right-censored observation. In general, lockdowns were implemented at the commune-level; however, at the beginning of the pandemic, some lockdowns were established at the greater administrative division level (i.e., province), which implies that a set of communes followed the same schedule. In this study, lockdowns were considered as commune-level.

For the longitudinal analysis, we studied several epidemiological factors collected at the first or third levels of the administrative division. The number of new asymptomatic and symptomatic cases, the number of patients in ICU, and the number of PCR exams performed were collected at the regional division level. At the commune level, we observed the number of active cases and the deaths per COVID-19 according to their residence. An active case was defined as a living person who met the definition criteria of a suspected case with a positive sample of SARS-CoV-2, whose date of onset of symptoms in the notification was less than or equal to 11 days, i.e., people capable of transmitting the infection. On the other hand, new symptomatic/asymptomatic cases correspond to new cases reported in a daily basis. All this information was considered as the number per 100,000 inhabitants. The original information used in this study was published by the Chilean Ministry of Science, Technology, Knowledge and Innovation and can be found on the GitHub repository available at https://github.com/MinCiencia/Datos-COVID19.

Based on the number of new asymptomatic and symptomatic cases and the number of PCR exams performed per region, we calculated a positivity index at the region level, which was expressed as the percentage of new cases relative to the number of PCR exams taken:

$$\text{positivity index}=\frac{\text{number of asymptomatic cases }+\text{ number of symptomatic cases }}{\text{number of PCR exams}}.$$

As the epidemiological information varies over time, we used two strategies to include them in the model. The number of deaths was included as a weekly sum during the period the commune was under lockdown, while the others were considered as daily average within each week.

### Predictors

Demographic and socioeconomic factors were considered as predictors for the time-to-event model. These consisted of population size (in scale of 100,000 inhabitants), number of immigrants (per 100,000 inhabitants), population density (number of people per km^2^), overcrowding (number of people over the number of households), a socioeconomic development index (SDI, ranging from 0 to 1), and a rural index of the communes (ranging from 0 to 1). For calculating SDI, which is performed by the Universidad Autónoma de Chile, different indicators are aggregated, including economy (monthly per capita income and poverty), education (average years of schooling), and housing and sanitation (good and acceptable housing material and sewerage or septic tank)^6^. For the calculation of the rural index, computed by the Ministry of Social and Family Development, it is considered the percentage of the rural population, the proportion of local employment occupied in primary sectors, and the population density. Then, an average of these three values was calculated, resulting in the rural index. Polanco^7^ has provided details about how to calculate such measure. Besides, we considered whether the commune held the regional or province city capital and whether a commercial airport or harbor exist in it. Apart from demographic and socioeconomic factors, we also included a binary covariate indicating if it was the first or second time that the commune was under lockdown.

### Statistical modeling

The two sources of information presented in this study are often analyzed separately through a survival analysis and a longitudinal analysis. However, in some situations, one may also be interested in the association between longitudinal measurements and the event of interest. In these cases, a joint approach is indicated, where information is shared between two or more models and each part provide relevant knowledge to the other. This procedure depends on the type of time-dependent covariates^8^. When this information is exogenous, i.e., variables whose cause is external to the model, an extended Cox model can be used^9^. On the other hand, when the longitudinal covariates are endogenous, i.e., variables that are changed or determined by their relationship with others, it is necessary to use a new class of models known as joint models^10^.

The idea behind joint modeling of longitudinal and time-to-event data is to couple a model for repeated measurements with a survival model to explain the event of interest. The most common joint model specification is to connect a mixed-effects sub-model fitted to describe the evolution of the longitudinal information with a proportional hazard sub-model fitted to the survival information. This approach had been limited to a single longitudinal and a single time-to-event outcome for a long time. However, a model with multiple longitudinal and/or multiple time-to-event outcomes can also be considered^11,12^. Thus, a joint model for $k$ longitudinal outcomes can be formulated as follows:

$$\left\{ \begin{array}{l}   y_{{ik}} \left( t \right) = m_{{ik}} \left( t \right)~ + ~\varepsilon _{{ik}} \left( t \right) = ~x_{{ik}} ^{T} \left( t \right)~\beta _{k}  + z_{{ik}} ^{T} \left( t \right)~b_{{ik}}  + ~\varepsilon _{{ik}} \left( t \right), \hfill \\   h_{i} \left( t \right) = h_{0} \left( t \right)\exp \left\{ {{{\gamma }}^{T} {\text{w}}_{{\text{i}}}  + {{~\alpha ~}}m_{{ik}} \left( t \right)} \right\}, \hfill \\  \end{array}  \right.  $$

where ${y}_{ik}\left(t\right)=({{y}_{i1}}^{T}(t), \dots , {{y}_{il}}^{T}(t))$ represents the *k*-variate vector of continuous longitudinal measurement for the $i$th commune at time $t$ with $k=1, \dots , l$. This vector is modeled by a mixed-effects sub-model, where ${\beta }_{k}$ denotes the regression coefficients associated with the design vector for the fixed effects ${x}_{ik}(t)$. Besides, ${z}_{ik}(t)$ denotes the design vector for the random ${b}_{ik}$ for the commune $i$. Finally, ${\varepsilon }_{ik}\left(t\right)$ represents the model error term. The longitudinal sub-model included fixed intercepts and random slopes. The joint model is completed with the time-to-event sub-model. In this case, the outcome is modeled by a proportional hazard. This kind of strategy focuses directly on the hazard function ${h}_{i}\left(t\right)$, considering the baseline hazard function, ${h}_{0}\left(t\right)$, and a second term that includes baseline covariates, ${\text{w}}_{\text{i}}$, and the true and unobserved value of longitudinal outcome for the commune $i$ at time $t$, which is denoted by ${m}_{ik}\left(t\right)$ and modeled by the longitudinal sub-model. Finally, α represents the association between the longitudinal and time-to-event outcome.

In this study, we were interested in investigating the time until a Chilean commune comes out of lockdown, which motivated the use of time-to-event sub-model. In addition, we wanted to add epidemiological information to the study; such information was obtained at the commune-level and over time during the follow-up period. Consequently, the most indicated strategy was to build a mixed longitudinal sub-model. Finally, the joint approach connected both parts, including the information obtained by the longitudinal model in the time-to-event model. Active cases, ICU patients, and deaths were logarithmically transformed for joint analysis, while the positivity index was handled as proportion.

### Model building process and model validation

The model was built in two stages. First, we fitted univariate joint models for each longitudinal factor (active cases, ICU patients, deaths, and positivity index). At this point, we identified that the association between the number of deaths and the duration of lockdown was not statistically significant, i.e., the p-value was higher than 0.05. Then, a bivariate joint model was fitted, considering pairs of the significant variables. Finally, a multivariate joint model was fitted; however, the association between ICU patients and the duration of lockdown was not statistically significant. Consequently, the bivariate model, including the number of active cases and the positivity index, was considered the final version.

The second stage was aimed to select the social and demographic covariates included in the bivariate joint model. To do so, we used the stepwise backward elimination approach, starting from a full model, which included all the predictors described in the previous section. Covariates with the highest p-values were removed from the model one at a time until all predictors were below the significance threshold (p-value ≤ 0.05). Finally, the joint model included two longitudinal information, number of active cases and positivity index, and one demographic and socioeconomic factor, overcrowding. All the statistical analyses were performed with R statistical software (version 4.1.0), with the level of significance set at 5%. Package joineRML was used to fit the joint model extended to multiple continuous longitudinal measures^11^.

## Results

### Descriptive analysis

Between March 25th and December 25th, 2020, 147 communes were under lockdown at least once and 19 (11.4%) twice, which resulted in a sample size (N) of 166. The lockdown duration was between 7 and 172 days, being 63 the average time. Of all communes, 16 (9.6%) are regional capitals, and 26 (15.7%) are province capital cities. In addition, there are commercial airports in 9 (5.4%) of them and commercial harbor in 10 (6%). Commune’s population size ranged between 1983 and 645,909 inhabitants, with a median population of 82,110 and median overcrowding of 2.73 people per household. Table 1 shows descriptive statistics for the outcome and predictors.

**Table 1:** Descriptive statistics of outcome and covariates considered in the model building process for the 147 communes included in the study (N = 166).

Variable	Mean	Median	Standard deviation	Range
**Outcome**				
Lockdown duration (days)	63	51	42	7–172
**Demographic and socio-economic covariates**				
Population (in scale of 100,000)	1.13	0.82	1.15	0.02–6.46
Population density (people/km^2^)	2289	122	4539	0–21,706
Overcrowding (people/households)	2.75	2.73	0.40	1.41–3.69
Immigrants (per 100,000)	2634	1098	3809	0–22,008
SDI (0–1 scale)	0.60	0.60	0.14	0.26–0.99
Rural index (0–1 scale)	0.41	0.41	0.16	0.04–0.79
**Longitudinal outcomes**				
Active cases (per 100,000)	186	150	156	0–2925
ICU (per 100,000)	8	6	5	1–18
Deaths (per 100,000)	399	275	340	0–1449
Positivity index (0–1 scale)	0.14	0.09	0.11	0.01–0.53

*SDI* socioeconomic development index, *ICU* intensive care units.

According to the United Nations, in 2019, 4.92% of the Chilean population was composed of immigrants, totaling almost one million people. Particularly in this study, only four of the 147 communes had no immigrants. On the other hand, the Chilean capital was the one with greater concentration of immigrants, 22,008 per 100,000 inhabitants. The rurality index of the communes also varied greatly: while some communes were entirely urban, others included almost 80% of rural areas. Something similar happens with SDI, which varied between 26 and 99%.

Regarding epidemiological characteristics, there was a great variation across the communes. The number of active cases ranged from zero up to 2925 cases per 100,000 inhabitants, while the number of people who entered the ICU varied between 1 and 18. There was also a great variation in the number of deaths per COVID-19, reaching 1449 deaths per 100,000 inhabitants. The positivity index varied between 1 and 53% depending on the commune and the week. More details about the continuous features considered in this study are displayed in Table 1.

### Joint model

The model building process started considering all predictors shown in Table 1. However, covariates with a p-value higher than the significance threshold were removed. Thus, population, population density, immigrants, SDI, rural index, number of ICU beds, and number of deaths were eliminated. The final model included overcrowding, number of active cases, and positivity index.

Results from the longitudinal sub-model showed that the number of active cases and the percentage of positivity decreased during the lockdown (Table 2). However, besides identifying this behavior, our primary goal was to know whether the number of active cases and positivity were associated with the duration of lockdowns. Thus, in the joint model, α indicates the change in the hazard for a unit change in the underlying subject-specific value of the longitudinal outcome, determining the strength of the association. Specifically, both longitudinal outcomes, number of active cases and percentage of positivity, and time-to-event outcome were significantly associated (${\alpha }_{1}$ and ${\alpha }_{2}$, respectively).

**Table 2:** Bivariate joint model.

Variable	Estimate	Standard error	p-value	HR (CI 95%)
**Longitudinal sub-model**				
Intercept	5.440	0.145	< 0.001	–
Active cases	− 2.740	0.287	< 0.001	–
Intercept	0.219	0.013	< 0.001	–
Positivity	− 0.406	0.036	< 0.001	–
**Time-to-event sub-model**				
Overcrowding	− 0.928	0.388	0.017	0.395 (0.185, 0.846)
Active cases	− 0.773	0.218	< 0.001	0.462 (0.301, 0.707)
Positivity	− 24.104	3.130	< 0.001	0.000 (0.000, 0.000)

Coefficient estimates, standard errors, and p-values for explanatory variables in both longitudinal and time-to-event sub-model, and hazard ratio (HR) estimates with their corresponding 95% confidence intervals (CI).

From a survival perspective, an increase on either active cases or positivity index implies a longer lockdown. The increment of one unit on the logarithm of active cases increases 53.7% the risk of staying under lockdown, i.e., a higher number of active cases implies longer lockdown. Similarly, for every additional percent unit of the positivity index, the risk of the lockdown being maintained goes up by 21.4% (HR = $exp\{-24.120*0.01\}$). Regarding demographic and socioeconomic factors, the joint model showed that overcrowding was significantly associated with the lockdown duration (p-value < 0.05), Table 2. For every additional unit of overcrowding, the risk of staying in lockdown goes up by 60.5% (HR = 0.395).

**Figure 1:**
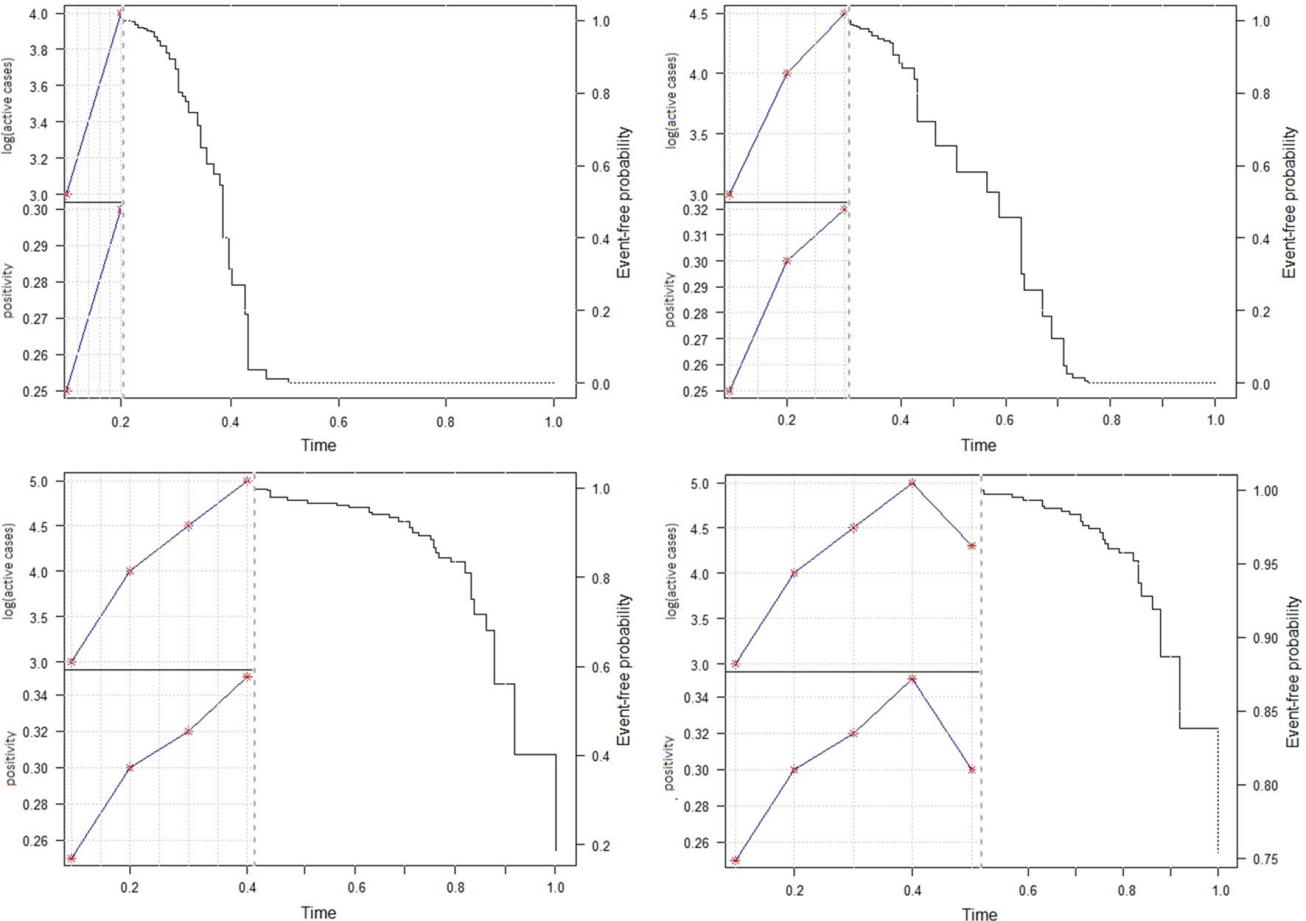
Dynamic predictions for commune of Santiago. The x-axis represents times. The y-axis of the left side represents the active cases in the logarithmic scale and the positivity. The stars represent the observed values and the solid line the fitted longitudinal trajectory. The y-axis on the right side represents the mean estimator of the predictions.

Joint model of longitudinal and time-to-event data is also a valuable tool to obtain predictions of survival probabilities. As detailed by Andrinopoulou et al.^12^, the predictions can be updated each time a new longitudinal measurement is available. Figure 1 shows predictions of the risk of lockdown being maintained for the commune of Santiago according to active cases and positivity observed at up to five. To do so, we set positivity as 0.25, 0.3, 0.32, 0.35, and 0.3. Similarly, active cases (in log scale) are considered as 3, 4, 4.5, 5 and 4.3.

### Reproducibility material

To facilitate the reproducibility of this study, both the data and the R code are provided in the supplementary materials, available at https://github.com/COVID0248/JM. Besides, details about the data sources are also available. Although we have not presented several details about joint models, we encourage our readers to look for more details about this modeling strategy. Asar et al.^13^ and Andrinopoulou et al.^12^ presented good tutorials focusing on how to use joint models in different contexts in epidemiology.

## Discussion

In clinical and epidemiological research, it is common to have longitudinal and time-to-event outcomes in the same study. Then, we have realized that similar situations also occur in other scenarios, such as COVID-19 lockdowns. In this study, we proposed a joint modeling framework to investigate more about Chilean lockdowns and their related factors. More studies using the joint modeling approach may be found in the literature. For instance, Chen et al.^14^ used a similar strategy for developing a dynamic risk prediction model for COVID-19 prognosis considering longitudinal measures. Lu et al.^15^ used a joint approach to study the association of oxygen saturation to the fraction of inspired oxygen ratio and time to death of patients diagnosed with COVID-19. However, to the best of our knowledge, no similar studies on the duration of lockdowns are available in the literature. Hence, this study aimed to investigate how demographic, socioeconomic, and epidemiological factors are associated with the duration of lockdowns in Chile. We used a multivariate joint model to explain these associations, providing valuable and practical information to support governmental decisions regarding local lockdowns. Apart from epidemiological reports (e.g., active cases and positivity index), we found that demographic and socioeconomic information (e.g., overcrowding) was also significantly associated with the duration of local lockdowns.

From an epidemiological point of view, it is essential to clarify that this study was not designed to determine whether the implementation of lockdowns at the commune level in Chile was an effective measure in terms of reducing contagion, but rather to explore whether the permanence of the communes under lockdown obeyed what we could call a “sanitary logic”. The variables considered by the authorities to keep a commune under lockdown in Chile are numerous and heterogeneous (e.g., reduction in the number of active cases, occupation of ICU beds, basic reproductive number, ability to trace and isolate cases, among others). Our study revealed that the decision made by the authorities followed what we previously called “sanitary logic”, because an increase in the number of active cases and positivity index reflects a worsening in the progress of the pandemic, which would merit keeping a commune under lockdown. Of particular interest is our finding regarding the variable overcrowding (i.e., number of people over the number of households), whose intensity also finished up as a factor associated with maintaining a lockdown. A possible explanation would be the following: the communes with the most significant overcrowding at the household level are communes in which there is a greater chance of intra-household contagion, thereby increasing the number of active cases and the positivity index in the commune, both being variables associated with the maintenance of lockdowns. Nevertheless, considering the complexity of the multivariable phenomenon under study, it is difficult to establish cause-effect relations, especially with design restrictions. Furthermore, factors such as mobility or traceability of cases, which we do not have information, could also affect the duration of the lockdowns. This would imply a potential bias in our results.

Finally, since many countries worldwide have established lockdowns, it would be interesting to compare our results with those obtained in other nations and thus evaluate their external validity. However, this is not a straightforward comparison once the term "lockdown" is not well defined. In fact, according to WHO, this term refers to large-scale physical distancing measures and movement restrictions, which indicates an unclear definition. Indeed, there are different adjectives used for the term, such as “total”, “full”, “hard”, “partial”, and/or “soft” lockdown suggesting also different degrees of restrictions. In addition, when reviewing literature and international media, we have found a lack of clear and consistent strategies for both entering and exiting lockdown. In the context of easing lockdowns, Han et al.^16^ analyzed the approaches taken by nine countries. They suggested that countries should base their decisions on a combination of epidemiological, social, economics, and demographic factors, which corroborates ours in spirit.

## Data Availability

All materials are available at https://github.com/COVID0248/JM.

## Code availability

R statistical software version 4.1.0.
